# Non-Conjugated-Industrially-Produced-Trans Fatty in Lebanese Foods: The Case of Elaidic and Linolelaidic Acids

**DOI:** 10.3390/nu13103664

**Published:** 2021-10-19

**Authors:** Maha Hoteit, Edwina Zoghbi, Alissar Rady, Iman Shankiti, Carla Ibrahim, Ayoub Al-Jawaldeh

**Affiliations:** 1 PHENOL Research Group (Public Health Nutrition Program-Lebanon), Faculty of Public Health, Lebanese University, Beirut 6573, Lebanon; m.hoteit@ul.edu.lb (M.H.); carla.t.ibrahim@outlook.com (C.I.); 2 Country Office for Lebanon, World Health Organization, Beirut 5391, Lebanon; zoghbi@who.int (E.Z.); Radya@who.int (A.R.); shankitii@who.int (I.S.); 3 Department of Nutrition and Food Sciences, Faculty of Arts and Sciences, Holy Spirit University of Kaslik, Jounieh P.O. Box 446, Lebanon; 4 World Health Organization Regional Office for the Eastern Mediterranean, Cairo 11371, Egypt

**Keywords:** industrially-produced trans fatty acids, Elaidic acid, Linolelaidic acid, traditional dishes, Arabic sweets, market foods, Lebanon

## Abstract

To determine Industrially-Produced Trans fatty acids (IP-TFAs) distribution of Lebanese traditional foods, especially regarding Elaidic acid (EA; 9t18:1) and Linolelaidic acid (LEA; 9t12t18:2), a mapping exercise was enrolled between January 2019 and April 2021 in which 145 food samples of three categories (traditional dishes, Arabic sweets, and market food products) were analyzed using Gas chromatography methods. Results showed that about 93% of the products tested in Lebanon, between 2019 and 2021, met the World Health Organization recommendations, while about 7% exceeded the limit. The mean level of the IP-TFAs Elaidic and Linolelaidic acid in most Traditional dishes (0.9%), Arabic sweets (0.6%), butter and margarine (1.6%), and market foods (0.52%) were relatively low compared with other countries. Despite that, the relative impact of IP-TFAs on heart diseases mortality in Lebanon is limited but unambiguously still substantial. The persistence of food products with high IP-TFAs levels threatens the health of Lebanese people. Fortunately, this problem is fairly easy to solve in Lebanon via proper legislation.

## 1. Introduction

The intake of Industrially-produced-Trans fatty acids (IP-TFAs) is associated with an increased risk of heart attacks and death from coronary heart disease (CHD) [1]. A 2% absolute increase in energy intake from IP-TFAs has been associated with a 23% increase in cardiovascular risk [2]. In 2018, IP-TFAs elimination was identified as one of the priority targets in the World Health Organization (WHO) 13th General Programme of Work, which guides the five-year work of WHO in 2019–2023 [3]. Also, in 2018, the REPLACE action package was launched to help countries removing IP-TFAs from their food supplies [4]. In addition, WHO released additional resources in 2019 to support country actions, including six implementation modules and a live policy tracking map—the TFAs Country Score Card 1—to monitor global progress towards the 2023 target [3]. In 2020, WHO established an indicator that records whether countries have adopted WHO best-practice policies for eliminating IP-TFAs [5]. Around fifty-eight countries have introduced laws to date that will protect more than 3 billion people from TFAs by the end of 2021 [3]. However, more than 100 countries have yet to act to eliminate TFAs from their national food supply and make the world TFAs free by 2023 [3]. The European Region has the largest number of mandatory TFA limits in place and has had the most policy progress of all WHO regions since 2019. Since Denmark’s effort (2004), Austria (2009), Iceland (2011), Hungary (2014), Norway (2014), Latvia (2018), Slovenia (2018) [6], and New Zealand (2008) have passed similar best-practice regulations [7]. Switzerland, one of the first countries in Europe to take legal action to restrict TFA, has a TFA limit in oils and fats (2008) [6]. The Eastern Mediterranean Region (EMR), as well as Lebanon, have witnessed rapid modernization in the last thirty years that has led to a dramatic transformation affecting people’s lifestyles and diets. The average intake of saturated fatty acids (SFAs) and IP-TFAs in EMR exceeded the WHO upper limits and was estimated to be 10.3% and 1.9% of total energy intake (EI), respectively [8]. The highest SFAs intake was reported in Djibouti, Kuwait, Saudi Arabia, Lebanon, and Yemen, while the highest intake of IP-TFAs was reported in Egypt and Pakistan [8]. According to recent national data, the proportion of coronary heart diseases (CHD) death due to IP-TFA intake is 9.4% (>0.5% energy) [5] and a high burden of NCDs, accounting for 91% of total annual deaths with CVDs responsible for 47% of total deaths [9] was observed in Lebanon. As a result, the urgent need for policy measures to protect cardiovascular health is more apparent than ever and presents a historic imperative to prioritize and invest in public health by adopting health-promoting policy measures, including industrially produced Trans fatty acids (IP-TFAs) elimination. Although limited data are available on IP-TFAs intake globally, a recent report estimated that the 2017 global market volume of partially hydrogenated vegetable oils (PHVO)—the main source of IP-TFAs in food—was approximately 13.6 million tones [10]. PHVO constitutes 25% to 45% of total fat [6]. Their removal from the global food supply could prevent up to 17 million deaths by 2040 and would be the first time an NCDs risk factor has been eliminated [11]. The most common non-conjugated IP-TFA in the human’s daily diet are 18-carbon fatty acids with one double bond in the 9-carbon transposition or two double bonds in the 9 and 12 carbon, called Elaidic acid (EA; 9t18:1) and Linolelaidic acid (LEA; 9t12t18:2) respectively [12]. EA and LEA were associated with various health problems [13]. EA, which is the *trans* form of oleic acid (OA, C18:1 *cis*), is the principal IP-TFA found in PHVO and margarine. EA intake resulted in significant hyperlipidemia, inflammation, and fatty liver alterations [14]. LEA is an omega-6 TFA (9E,12E-9t12t18:2), principally discovered in foods with fried or high-heat cooking or PHVO [15]. It was suspected to enhance the adipogenic differentiation favoring obesity [15]. Moreover, LEA appeared to be potentially more detrimental than EA and LEA contributed to higher risks of sudden cardiac death compared with other TFAs [16]. Because IP-TFAs increases the risk of heart disease and are estimated to cause more than 500,000 deaths per year [3] and based on the WHO recommendation that IP-TFAs intake should not exceed 1% of total daily energy intake (equivalent to less than 2.2 g/day in a 2000-calorie diet), providing baseline information on dietary sources of IP-TFAs in Lebanon is a crucial stepstone to reduce the risk of death and hospitalization by CVDs and is one of the strategic interventions under the area of prevention and reduction of risk factors in the Regional Framework for Action on NCDs [17]. To our knowledge, this is the first national study that assesses the content of EA and LEA in food. The main objectives of this article are to:

Assess IP-TFAs levels, mainly EA and LEA in frequently consumed traditional dishes, Arabic sweets, processed foods, butter, and margarines in Lebanon.

Review of the findings retrieved from online databases on dietary sources of IP-TFAs in Lebanon and compare them with other countries.

Establish a steppingstone for required policies and regulations to mandate limits of IP-TFAs levels in foods imported or produced locally.

## 2. Materials and Methods

### 2.1. Food Sampling

**Figure 1:**
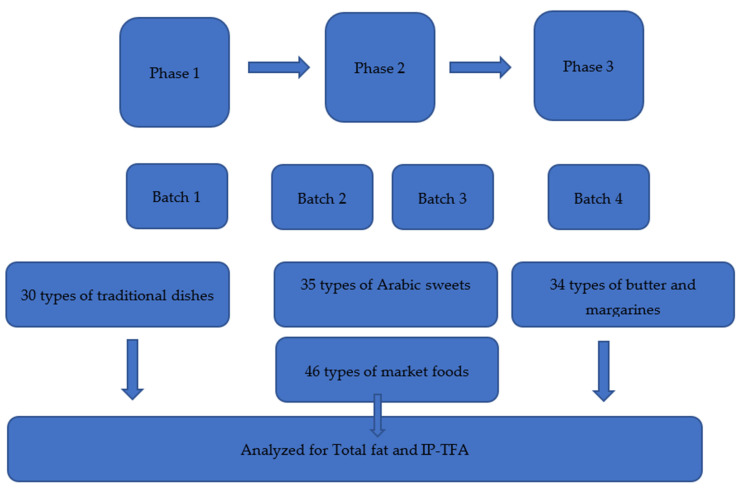
Graphical scheme of the current study.

A series of samples collections were conducted over the last two years. The 2019 samples collection, conducted in November 2019, was not centrally coordinated at the capital city Beirut but instead pooled data from five separate sources from the five main governorates in Lebanon (Beirut, Beqaa, Tripoli, Saida, and Mount Lebanon). In this sample collection, we collected thirty types of traditional dishes. Traditional composite dishes are defined as dishes consumed at main meals (i.e., lunch or dinner), containing ingredients from at least three of the five main food groups and requiring preparation using culinary skills [18,19,20]. A total of 30 traditional composite dishes were identified as most frequently consumed and hence were included for analysis. The names of the food dishes were reported in the current analysis considering the most familiar name used for the dish at a national level with respect to its ingredients. The ingredients of these traditional dishes were described in Hoteit et al. [18,19,20], and the food samples were collected from five different central kitchens in the 5 governorates listed above. The central kitchens were randomly chosen based on (1) their specialties in cooking homemade dishes, (2) their popularity in the area, (3) their implications in social entrepreneurship and women empowerment (e.g., household women who cook for these central kitchens). Consequently, the food samples were classified into 5 strata, per governorate area [20]. The samples were identified according to their frequency of consumption [21,22] and selected for IP-TFAs analysis mainly for two non-conjugated fatty acids (EA and LEA). In contrast, the subsequent samples collections 2020, conducted in April, were centrally coordinated at Beirut having the broadest coverage in terms of products selected and had a sample of thirty-five types of Arabic sweets and forty-six types of market food products. The full methodology of food list identifications and food sampling is described elsewhere [18,19,20]. On the other hand, the 2021 sample collections, conducted in March, were nationally coordinated, with a coverage of 34 available types of butter and margarines purchased from all the Lebanese markets. Lot numbers were checked to ensure that each unit belonged to a different lot. The samples were stored, labeled, and analyzed before expiry dates. Samples were selected to include all types of butter and margarines in Lebanon. The analyses were carried out in duplicate for each sample. Thus, a composite sample from each type of food, according to each governorate, was prepared and analyzed. To further interpret current levels of IP-TFAs in Lebanese foods, product categories were compared with similar products found in other countries. A graphical scheme for the whole study is shown in Figure 1.

### 2.2. Laboratory Analysis Protocol

Around 500 g of each sample was mashed, then analyzed, and the remaining samples were kept frozen at −18 °C for further analysis. The fatty acid profile was measured using gas chromatography. The IP-TFA analysis method was selected considering guidance from the technical committee at the Industrial Research Institute laboratories in Beirut and following standardized protocols. The Association of Official Analytical Chemists (AOAC) methods were used for the analysis of nutrients in food matrices [23].

Soxhlet extraction (Total fatty acids extraction):

The Roese–Gotlieb method was used in the investigation of the fat content [24]. Between 1–2 g of the dried food sample was filtered using a piece of filter paper. Later, it was wrapped and introduced into the soxhlet thimble. To avoid sample spilling, a cotton plug was placed at the top of the thimble. The soxhlet apparatus was assembled, and light petroleum, hexane, heptane, diethyl ether, or cyclohexane was used. The extraction was performed overnight. If solids from the thimble or sample were found in the solvent extract, a filtration before evaporation into another tarred flask or beaker was performed. Then it was dried to a constant weight, which was attained when successive 1 h drying periods showed additional loss of less than 0.05 fat. The percentage (%) fat was equal to (g) fat × 100 g sample.

Fatty Acid Profile (saturated, unsaturated, trans):

The extracted fat of the sample that was obtained during fat determination was used to analyze the fatty acid profile. Between 200–500 mg of the lipid sample was placed in a boiling flask with chips; then, 5 mL of 0.5 M methanolic KOH was added. Esterification was performed by boiling under reflux for 3–5 min. An addition of 15 mL of esterification reagent (2 g NaNH4 + 60 mL methanol + 3 mL conc sulfuric acid) through the condenser was performed, and then the sample was boiled for 15 min. After cooling, there was an addition of 50 mL of distilled water and 25 mL of the solvent. The organic layer was isolated by means of a separatory funnel. Finally, the solvent layer was washed twice with distilled water.

Chromatographic analysis:

- Column: fatty acid methyl esters (FAME) length: 30 m, 0.32 ID
- Injection volume: 1 µL
- Injector temperature (PTV): injection: 60 °C for 0.1 min
- Transfer: ramp 10 °C/min to 270 °C, 1 min hold
- Carrier flow (He): 2.0 mL/min
- Split flow: 20 mL/min (split ratio: 20)
- Detector temperature (flame ionization detection (FID) 280 °C
- Detector gases flows: Air 350 mL/min, Hydrogen 32 mL/min, Make-up (N2) 30 mL/min

Oven Program:

- Initial temperature: 100 °C, hold 3 min
-Ramp 1: 10 °C/min to 200 °C, hold 3 min
-Ramp 2: 10 °C/min to 250 °C, hold 5 min

Using the chromatograph software Chromquest 4.2.34, USA, an integration of the areas under the peak was detected under the standards peaks. A calculation as percentage areas was done. The sum of trans fatty acids was calculated accordingly [24]. TFAs isomers were later on detected through SP-2560 100 m capillary column (180 °C isothermal, H2 at 1.0 mL/min) [23].

Statistical tests:

The study variables were presented as continuous variables and listed as reported values per 100g. Means and standard deviations were calculated for the group of traditional dishes, Arabic sweets, and other market products. T-test was used to compare the mean content of the group of foods in terms of EA and LEA. A *p*-value less than 0.05 indicates a statistically significant difference. Statistical analysis was conducted on IBM SPSS Statistics for Mac, Version 24, USA.

## 3. Results

### 3.1. Trans Fatty Acid Acids in Frequently Consumed Traditional Dishes

The mean levels of total IP-TFAs (total of Elaidic acid and Linolelaidic acid) in the tested traditional dishes was equal to 0.9% ± 0.62 and ranged from less than 0.1 to 2.8 g/100 g of total fat except for the dishes *Riz a dajaj* and *Shawarma Lahma* in which total IP-TFA exceeded 2% of the total fat (Table 1). The comparison between the mean values of the IP-TFA (EA and LEA) in the traditional dishes tested shows that EA was significantly higher than LEA in all traditional dishes (*p*-value = 0.00). Per each governorate, the mean level of total IP-TFAs in all dishes was 0.78 ± 0.65% in Mount Lebanon (range: <0.1–2.1%), 1.1% ± 0.6 in Beqaa (range: <0.1–3%), 1 ± 2.4% in Beirut (range: <0.1–11.2%), 1.2 ± 0.9% in Tripoli (range: <0.1–3.2%) and 0.8 ± 0.4% in Saida (range: <0.1–2%) (Data not shown).

### 3.2. Trans Fatty Acid Acids in Frequently Consumed Arabic Sweets

The average of the total IP-TFAs in all samples of Arabic sweets was 0.6 ± 0.3%, predominantly from the EA type. (Table 2). Among 35 samples of Arabic sweets, none exceeded 2% as total IP-TFA in 100 g of total fat. The comparison between the mean values of the IP-TFA (EA and LEA) in the Arabic sweets tested shows that EA was significantly higher than LEA in Arabic sweets (*p*-value = 0.00).

### 3.3. Trans Fatty Acid in Market Foods

#### 3.3.1. Cereals and Breads Group

In the group of cereals and breads, the mean level of total IP-TFAs was less than 2% of total fat except for *pain au lait (total IP-TFAs: 3.8%)*, which is usually prepared from wheat, milk, and butter or ghee to be consumed frequently by children as a sandwich (Table 3 and Table 4).

#### 3.3.2. Butter and Margarines

Particular attention was given to the margarine group as it is used as an ingredient and therefore amongst the main sources of IP-TFAs in processed foods. The average of total IP-TFAs in 18 margarines used frequently in Lebanon was 2.4 ± 0.4% (Table 3) with a range between <0.1% and 11.8% (Data not shown). The dominant IP-TFA was EA in almost all these products (Table 3). Within the group of butter, none of the samples exceeded 2% of total fat. The average of total IP-TFAs in the butter and margarines group was 1.6 ± 0.6% of total fat in which EA predominates in these products. Generally, the level of total IP-TFAs in cooking oils, Halawa and Tahina was negligible (Table 3 and Table 4).

#### 3.3.3. Snacks and Processed Foods

As for the group of biscuits, doughnuts, and cakes group, negligible amounts of IP-TFAs were found in these products (Average: 0.5% ± 0.2) (Table 4). On the other hand, the unlabeled English cake (chocolate flavor) had an apparently high amount of total IP-TFAs (2.6% in the total fat) in which EA was dominantly available (Table 3). Despite being unable to discuss the fat type used in unlabeled samples, based on this data, partially hydrogenated fats were certainly present in high amounts.

The data on chocolate products presented an amount of 1.3% ± 0.3 as total IP-TFAs (Figure 1), except for the case of wafer-coated chocolate originally manufactured in Lebanon which contains a level of 6.5% (Table 3).

According to Table 3 and Table 4, it appears that all samples of potato chips, nuts, seeds, coffee, instant coffee, and packed tuna contained low amounts of total IP-TFAs that are below 2% of total fat. When comparing the mean values of the IP-TFA (EA and LEA) in the market foods, EA and LEA didn’t show any significant difference (*p*-value = 0.16).

## 4. Discussion

Industrially-Produced Trans fatty acid content in frequently consumed foods in Lebanon compared with different countries.

The available data, the first of its kind in Lebanon, demonstrate that categories with the highest IP-TFAs levels included *Riz a dajaj*, *Shawarma Lahma*, *Pain au lait*, *English cake*, *Chocolate wafers, and margarines.* About 93% of the products tested in Lebanon, between 2019 and 2021, met the WHO recommendations (less than 2% of Trans fatty acid in total fat), while about 7% exceeded the limit. As per Table 1, Table 2 and Table 3, all in all, EA was dominant in almost all the analyzed samples and its higher amount indicates that hydrogenated oils were a major contributor in the processing of food products or baking and cooking meals. In comparison to other countries all over the globe, a broad range of EA was observed in many food products (Table 4). For instance, the mean level of EA in Baklava (0.2%) was relatively low in our study in comparison with the content of EA in Baklava in Iran (2.5%) [25]. Furthermore, our findings showed that the mean levels of EA in cakes (2.6%) was much lower than the content of EA found in cakes in France (18.5–25.6%) [26], Iran (6.95–18%) [25,26,27], Poland (7.95%) [28], India (1.92–3.93%) [29], and higher than EA cake content tested in Lebanon in 2015 (1.7%) [30], Korea (1.36%) [31], Turkey (0.37–1.43%) [32], New Zealand (0.9%) [7], and Malaysia (<0.001%) [33] (Table 4). In addition, the mean levels of EA in biscuits in Iran (9–12.86%) [27], Lebanon 2015 (3.7%) [30], Poland (2.81%) [28], Korea (2.4%) [31], New Zealand (0.9%) [7], and Germany (0.18%) [34] were higher than our results (0.1%), except for Malaysia (<0.001%) [33] and India (0.01%) [29] (Table 4). As for the breakfast cereals, the mean level of EA in our study (0.1%) was much lower than in France (28.9–32.4%) [26] and Korea (0.5–6.75%) [31], and higher than in the UK (0.03%) [35] and Malaysia (<0.001%) [33] (Table 4). Moreover, our findings showed that the mean level of EA in chocolate wafers were six times more than EA content in chocolate wafers in Malaysia [33]. As for the butter, the New Zealand [7] and Costa Rican butter [36] contained five times more EA, and the Pakistani butter [37] contained three more times EA, compared with our results (Table 4). However, the butter in UK, Germany, and Iran contained 0.22% [35], 0.23% [34], and 0.3% [27] EA respectively; this is lower than the content of EA tested in our study (0.6%) (Table 4). Also, Table 4 showed that the margarines in Slovenia contained the highest content of EA (34.63%) [38] compared to our findings (2.2%) and other countries. As for the EA content in chips, Iranian chips showed the highest level of EA (10%) compared to our results (0.1–0.3%) and other countries [27] (Table 4). On the other hand, the results of LEA in the food products tested in our study and those in other countries are available in Table 4.

### 4.1. Comparison between Lebanese Market Basket Investigation and Other Global and Regional Market Investigations

According to many studies, there was an impact of TFAs labeling on reducing the burden of CVDs due to TFAs [39]. According to an unpublished study conducted by our team, 32% only of the products available in the Lebanese markets reported TFAs on their labels (Data not shown). Our finding came to hand by hand with Kamel et al. [40], in which 181 food products were sampled from local supermarkets in Saudi Arabia and showed that one-third of the products mentioned TFAs on the nutrition label. Moreover, while the majority of the investigated samples in our project had low levels of TFAs, up to 14 g of TFAs per 100 g of food was observed in certain oils and fats sold at the Lebanese markets. Our findings, concerning the range of TFAs in-market products, were relatively low compared with the market investigations published in Stender et al. (2019–2020) [41,42].

### 4.2. Investigation of the Country of Origin of Imported Food Products in Lebanon

Lebanon imported its food products from France ($107,957), Germany ($98,250), Turkey ($97,015), United Kingdome ($75,571), Italy ($70, 571), Argentina ($69,989), Saudi Arabia ($64, 332) and United States ($57,785). In addition, the main importation sources of butter, oils, and fats are Denmark, Netherlands, France, Belgium, Ukraine, New Zealand, United Kingdom, and Argentina [43]. According to the nutrition labels of tested butter and margarines, the country of origin from which all the butter and margarines were imported to Lebanon were Turkey (*n* = 5), Egypt (*n* = 4), Malaysia (*n* = 3), Saudi Arabia (*n* = 1), Sri Lanka (*n* = 3), UAE (*n* = 1), Netherland (*n* = 2), Belgium (*n* = 3), France (*n* = 4), Italy (*n* = 1), Ukraine (*n* = 1), Germany (*n* = 2), and Denmark (*n* = 2). Among all these countries, 33 percent (five countries over 15) are implementing mandatory national limits and adopting monitoring mechanisms for mandatory of TFAs limits. On the other hand, in the remaining countries, the best-practice TFAs policy passed but was not yet in effect [5]. Lebanon, long considered a middle-income country, is rapidly sinking into poverty as it faces a triple shock from the unprecedented economic crisis, the impact of COVID-19 on employment and public health, and the consequences of Beirut port explosions. Despite that, the actual relative impact of IP-TFAs exposure on heart disease mortality in Lebanon is limited, but unambiguously still considerable. The findings in our report highlight the importance of controlling the importation of food products from countries controlling IP-TFAs levels in food to avoid sinking Lebanese markets with IP-TFAs rich food products [44], both of which are often ultra-processed, unhealthy, and rich in IP-TFAs. Therefore, this population group is at higher risk of IP-TFAs-attributable CVDs.

### 4.3. Limits, Advantages, and Future Directions

This study presents some limitations. First, there are many challenges facing the laboratories in Lebanon concerning the testing of IP-TFA, and the lack of standards limits testing other forms of isomers. Second, the food products compared between regions were compared in terms of food groups and not in terms of brands. Moreover, the comparison between traditional dishes or Arabic sweets omits the cooking preparations and ingredients. Third, in the current study, the WHO technique was followed to test the IP-TFA levels in foods tested, however, this was not always reported in many other countries.

Despite these limitations, this study, the first of its kind in Lebanon, should provide the impetus for continuous comprehensive analysis of IP-TFA levels in foods in the regional and national kitchens and markets and the adaptation of the approaches for curbing the health hazards associated with IP-TFA consumption.

## 5. Conclusions

For the first time in Lebanon, a database on IP-TFA, mainly EA and LEA content in traditional dishes and market products is available and ready to be used by health care providers. There is more than enough convincing evidence that a high IP-TFAs, mainly EA and LEA intake is detrimental to cardiovascular health. Fortunately, this problem in Lebanon is fairly easy to solve via proper legislation. Despite the poorness of Lebanese dishes in IP-TFAs, however, the persistence of food products with high IP-TFAs levels in Lebanon means that subgroups of the Lebanese population, mainly vulnerable and food-insecure people, are threatened by high levels of IP-TFAs due to frequent consumption of risky products. The inauguration and implementation of policies to curtail IP-TFAs in Lebanon may therefore be legitimized, and such efforts should underline added fats and packaged foods. The economic crises in Lebanon pushed the Lebanese people to select cheap oils, including butter and margarines instead of vegetable oils. Thus, it appears reasonable that the Lebanese government and ministries should strive to raise public awareness about the issue and lobby for implementing anti-IP-TFAs laws either on the level of national industries or, on the level of food products importation.

## Figures and Tables

**Table 1:** Total fat in 100 g of edible portions, total IP-TFAs and IP-TFAs (EA and LEA) in 100 g of fat of frequently consumed traditional dishes among all Lebanese governorates.

			IP-TFAs in 100 g of Total Fat	
Dish	Total Fat (g) in 100 g	Total IP-TFA * per 100 g of Total Fat	Trans-C18:1n9t (Elaidic Acid)	Trans-C18:2n6t (Linolelaidic Acid)
Baba ghanouj	9.44	0.74	0.66	0.08
Batata mahchi	1.24	1.98	1.92	0.06
Borgul bil banadoura	5.02	0.48	0.34	0.14
Chichbarak	4.62	0.98	0.86	0.12
Falafel	11.70	0.36	0.32	0.04
Fatayer sabanikh	11.16	0.18	0.12	0.04
Fattat Hommos	7.04	0.66	0.58	0.08
Fattoush	2.94	0.8	0.5	0.3
Foul moudamas	3.48	0.46	0.38	0.08
Hindbe bil zet	10.70	0.18	0.18	0
Hommos bi tahini	6.44	0.38	0.24	0.14
Kafta wa batata	6.32	1.28	1.18	0.1
Kebba bil sayniya	6.40	0.86	0.74	0.12
Koussa mahchi	2.42	1.26	1.1	0.16
Lahm bil ajin	8.96	0.34	0.22	0.12
Loubia bil zet	5.68	0.52	0.46	0.06
Malfouf mahchi	2.12	1.1	1.02	0.1
Moujadara	5.80	0.36	0.36	0
Moghrabia	3.94	0.86	0.76	0.1
Mousaka batinjan	6.58	0.5	0.34	0.16
Riz a dajaj	5.42	2.82	2.66	0.16
Riz bi lahma	6.52	0.82	0.78	0.04
Sayadia	6.48	0.22	0.18	0.04
Shawarma dajaj	6.94	0.24	0.16	0.08
Shawarma lahma	8.28	2.24	2.08	0.16
Tabboula	4.24	0.38	0.26	0.12
Warak enab	3.98	1.24	1.06	0.18
Yakhnat Bamia	5.42	1.24	1.02	0.22
Yakhnat Fassoulia	3.90	0.76	0.64	0.12
Yakhnat Mouloukhia	4.28	1	0.8	0.2

* This represents the sum of EA and LEA only.

**Table 2:** Total fat in 100 g of edible portions, total IP-TFAs and IP-TFAs (EA and LEA) in 100 g of total fat of frequently consumed Arabic sweets.

			IP-TFAs in 100 g of Total Fat	
Name	Total Fat (g) in 100 g	Total IP-TFAs per 100 g of Total Fat *	Trans-C18:1n9t (Elaidic Acid)	Trans-C18:2n6t (Linolelaidic Acid)
Baklava Mixed	23.45	0.25	0.2	0.05
Baklava Mixed Light	20.5	0.3	0.3	0
Halawat El Jiben	8.95	1.2	1.05	0.15
katayef Kashta	6.65	0.9	0.65	0.25
Kounafa bil jiben	12.25	0.4	0.25	0.15
Maakaroun	12	0.1	0.1	0
Maamoul Tamer	17.4	0.4	0.25	0.15
Maamoul mad Kashta	10.65	0.4	0.25	0.15
Maamoul mad joz	19.2	0.45	0.4	0.05
Maamoul joz	21.5	0.85	0.75	0.1
Mafrouka Kashta	13.25	0.4	0.2	0.2
Mafroukeh fostok	10.6	0.6	0.5	0.1
Moushabak	20.1	0.4	0.4	0
Nammoura	5.9	1.5	1.3	0.2
Osmaliya	16.25	0.5	0.4	0.1
Saniora	23.8	1.15	0.85	0.3
Sfouf	12.45	1.45	1.2	0.25
Barazik	16.5	0.5	0.5	0
Boundoukia	19.5	0.3	0.3	0
Daoukia	14.8	0.4	0.3	0.1
Foustoukia	20.4	0.4	0.4	0
Ghourayba	0.325.8	0.6	0.4	0.2
Ish el bulbul	25.1	0.2	0.2	0
kallaj kashta	9.6	<0.1	-	-
Karabij joz maa crema	18.8	0.4	0.2	0.2
kounafa kashta maa kaak	10	0.4	0.1	0.3
Maakroun wa Moushabak	13.7	<0.1	-	-
Maamoul fostok	19.1	0.7	0.5	0.2
Madlouka	11.5	0.6	0.5	0.1
Moufattaka	20.7	<0.1	-	-
Mouhallabiya	4	0.5	0.1	0.4
Riz bil Halib	4.4	<0.1	-	-
Shaaybiyat	16.1	<0.1	-	-
Ward el sham	14.2	0.5	0.5	0
Znoud El sitt	12.3	<0.1	-	-

* This represents the sum of EA and LEA only.

**Table 3:** Total fat in 100 g of edible portions, total IP-TFAs and IP-TFAS (EA and LEA) in 100 g of total fat of Market food products collected from Lebanese markets.

			IP-TFAs in 100 g of Total Fat	
Product	Total Fat (g) in 100 g	Total IP-TFA per 100 g of Total Fat	Trans-C18:1n9t (Elaidic Acid)	Trans-C18:2n6t (Linolelaidic Acid)
Arabic Bread-White	2.3	<0.1	-	-
Arabic Bread-Whole wheat	4	<0.1	-	-
Baguette	0.5	<0.1	-	-
Biscuits Chocolate Quinoa	13.4	0.1	0.1	-
Biscuits Digestive	17.1	0.3	0.1	0.2
Biscuits Digestive Light	13.8	0.3	0.1	0.2
Biscuits with cream	15.5	<0.1	-	-
Breakfast Cereals	2.1	<0.1	-	-
Breakfast Cereals-Chocolate	2.4	0.3	0.1	0.2
Butter (*n* = 17 samples)	80	0.8	0.6	0.2
Cake with Cream	16.1	<0.1	-	-
Chocolate Dark	33.6	<0.1	-	-
Chocolate Milk-1	36.6	0.1	0.1	-
Chocolate Milk-2	35	<0.1	-	-
Coffee without cardamon	16.8	0.2	0.1	0.1
Coffee with cardamon	17.7	0.3	0.1	0.2
Corn Oil	100	<0.1	-	-
Croissant Zaatar-1 (cheap)	16.1	0.7	0.4	0.3
Croissant zaatar-2 (expensive)	22.5	0.1	0.1	
De-hulled Pumpkin Seeds	50.6	0.6	0.3	0.3
De-hulled Sunflower Seeds	52.5	0.7	0.3	0.4
Doughnuts	19.6	0.5	0.5	-
English Cake-Chocolate	18.6	2.6	2.6	-
Margarines (*n* = 18)	100	2.4	2.2	0.2
Halawa	25.5	0.4	0.4	-
Halawa Light	29.9	1.3	1.1	0.2
Hot Chocolate Powder	5.4	0.3	0.3	-
Instant Coffee	10.8	0.2	0.2	-
Kaak asrouni **	1.5	Tr	-	-
Kaak debes and Cacao ***	11.9	0.3	0.2	0.1
Kaak korshalli ****	6.9	0.5	0.5	-
Mixed Kernels	53.6	<0.1		-
Mixed Nuts	25.7	0.3	0.2	0.1
Olive Oil	100	<0.1	-	-
Pain au Lait	3.8	2.7	2.7	-
Petit Fours-1 (cheap)	25.6	0.2	0.2	-
Petit Fours-2 (expensive)	29.6	0.2	-	0.2
Potato Chips-1	29.9	0.1	0.1	-
Potato Chips-2	15.4	0.3	0.2	0.1
Potato Chips Light-1	26.9	0.1	0.1	-
Potato Chips light-2	22.9	0.3	0.3	-
Sunflower Oil	100	<0.1	-	-
Tahina	59.4	0.1	-	0.1
Tuna Packed in Oil	6.8	0.3	0.1	0.2
Tuna Packed in Water	0.5	0.6	0.6	-
Wafer-Chocolate-1	21.7	<0.1	-	-
Wafer-Chocolate-2 (manufactured in Lebanon)	24.2	6.5	6.2	0.3

** Kaak Asrouni: type of Lebanese street bread. *** Kaak Debes and Cacao: Cacao cookies with molasses. **** Kaak korshalli: toast bagel (elongated shape).

**Table 4:** Industrially-Produced trans fatty acids (EA and LEA) in 100 g of total fat per food groups among different countries.

	IP-TFAs	
Countries	Trans-C18:1n9t (Elaidic Acid) (%)	Trans-C18:2n6t (Linolelaidic Acid) (%)
France	Cake: 24.43 Cereals: 28.9 Roasted bread: 33.1 Toasted bread: 25.8 Bread: 30.3 Cookies 38.9	-
France	Cake: 18.5	-
New Zealand	Margarines and table spreads (low trans): 0.1 Margarines and table spreads: 12.3 Margarine/butter blends: 8.3 Butters: 5.2	Margarines and table spreads (low trans): 0.1 Margarines and table spreads: 1.3 Margarine/butter blends: 1.6 Butters: 1.7
Spain	Spanish margarines: 8.17	Spanish margarines: 0.49
Bulgaria	Imported margarines: 8.4 Bulgarian margarines: 1.12	-
Turkey	Margarine tub: 3.85 Margarine stick: 16.88	Margarine tub: 0 Margarine stick: 2.09
Korea	Breakfast cereal: 6.75 Cream-filled biscuit: 15.57 Cream-stuffed cake: 20.96 Canned coffee: 2.3	Breakfast cereal: 0.25 Cream-filled biscuit: 0.43 Cream-stuffed cake: 0.66 Canned coffee: 0.3
New Zealand	Biscuits and cakes: 0.9 Margarines/spreads: 4.9 Chocolate: 1.1 Snack bars: 0.4 Pies and pastry: 3.7 Partially cooked chips/wedges: 2.5	Biscuits and cakes: 0 Margarines/spreads: 0.1 Chocolate: 0 Snack bars: 0.1 Pies and pastry: 0.4 Partially cooked chips/wedges: 0.4
Pakistan	Margarines: 7.89 Butter: 3.82	Margarines: 0.45
Turkey	Margarines and shortenings: 10.55	-
Canada	Tub margarines: 3.4 Print margarines: 5.5	Tub margarines: 0.1 Print margarines: 0.3
Costa Rica	Corn oil: 0.35	Corn oil: 0.07
	Sunflower oil: 0.28	Sunflower oil: 0.09
	Olive oil: 0.26	Olive oil: 0
	Margarines: 10.15	Margarines: 0.35
	Butter: 5.1	Butter: 0.23
	Mixed nuts: 0.2	Mixed nuts: 0
		Mayonnaise: 0.02
	Canned tuna (oil): 0.54	Canned tuna (oil): 0.08
	Canned tuna (water): 1.07	Canned tuna (water): 0
	Nondairy coffee creamer: 30.84	Nondairy coffee creamer: 1.15
Korea	Breakfast cereal: 0.5 Cream-filled biscuit: 2.4 Cream-stuffed cake: 1.36 Canned coffee: 2.3	Breakfast cereal: 0.3 Cream-filled biscuit: 0.25 Cream-stuffed cake: 0.26 Canned coffee: 0.7
Pakistan	Margarines: 19.48	Margarines: 0.49
Brazil	Regular dark Chocolate: 0.078 Regular chocolate: 0.075	-
Germany	Margarines/spreads: 0.2 Shortenings/cooking fats: 0.51 Doughnuts: 2.07 Chocolate products: 0.44 Biscuits: 0.18 Instant coffee products: 0.36 Butter: 0.23	-
Mexico	Spreadable margarines: 4.73 Stick margarines: 7.4	Spreadable margarines: 0.39 Stick margarines: 0.94
Turkey	Potato crisps: 0.13 Corn crisps: 0.24 Cocoa cakes: 0.37 Chocolate cakes: 0.55 Cream cakes: 0.78 Fruity cakes: 1	Potato crisps: 0.15 Corn crisps: 0.16 Cocoa cakes: 0.11 Mosaic cakes: 0.05 Chocolate cakes: 0.08 Cream cakes: 0.24 Hazelnut-cocoa cakes: 0.14 Fruity cakes: 0.09
India	Biscuit: 0.01 Pastry: 0.85 Cake: 1.92 Bread: 0.18 Bun: 1.31	Biscuit: 0 Pastry: 0 Cake: 0.04 Bread: 0.007 Bun: 0.03
Iran	Cakes: 18 Cream biscuits: 12 Simple biscuits: 9 Simple chocolates: 5 Potato chips: 10 Margarine: 3.2	Cakes: 0 Cream biscuits: 2 Simple biscuits: 2 Simple chocolates: 0 Potato chips: 4 Margarine: 0.9
UK	Breakfast cereal products: 0.03 Margarine, hard block: 0.05 Potato chips, takeaway: 0.97 Potato chips, fine cut, takeaway: 0.08 Potato chips, oven baked: <0.02 Potato snacks and corn snacks: 0.08 Confectionery, non-chocolate: 0.05 Confectionery, chocolate: 0.08 Butter, spreadable: 0.22	-
Malaysia	Cakes: <0.001 Doughnuts: <0.001 Croissants: <0.001–0.02 White bread: <0.001 Whole grain bread: <0.001 Buns: <0.001 Cream crackers: <0.001–0.33 Chocolate biscuits: <0.001 Potato chips: <0.001–0.87 Chocolate bars: <0.001 Chocolate wafers: <0.001–0.38 Olive oil: 0.79 Blended oil (canola, soybean and olive): 0.82 Soybean oil: 1.76 Palm oil: 1.79 Corn oil: <0.001 Coco-coated cereal: 1.57 Corn cereal: <0.001 Cereal beverages: <0.001	Cakes: <0.001 Doughnuts: <0.001 Croissants: <0.001 White bread: 3.12 Whole grain bread: <0.001 Buns: <0.001–1.21 Cream crackers: <0.001 Chocolate biscuits: <0.001–0.02 Potato chips: <0.001–1.02 Chocolate bars: <0.001–0.54 Chocolate wafers: <0.001 Olive oil: <0.001 Blended oil (canola, soybean and olive): 3.24 Soybean oil: 4.06 Palm oil: <0.001 Corn oil: 2.13 Coco-coated cereal: <0.001 Corn cereal: 4.82 Cereal beverages: <0.001–6.60
Iran	Liquid frying oils: 0.08 Solid frying oils: 1.26	Liquid frying oils: 0.01 Solid frying oils: 0.03
Saudi Arabia	Margarines and shortenings: 5.43	Margarines and shortenings: 1.49
Iran	Margarines: 5.99	Margarines: 0.66
Iran	Biscuit: 12.86 Cake: 6.95 Shortcake: 3.38 Donuts: 3.29 Bread tan: 2.99 Baklava: 2.5 Chocolate: 1.24 Chips: 0.61 Snack: 0.52	-
Iran	Edible oils: 0.07 Margarines: 5.3	-
India	Cakes: 3.93	Cakes: 2.82
Lebanon	Cakes: 1.7 Biscuits: 3.7 Croissant: 2.7 Wafers: 5.6	Cakes: 0.1 Biscuits: 0.1 Croissant: 0.1 Wafers: 0.1
Slovenia	Margarines and shortenings: 34.63	Margarines and shortenings: 21.38
Serbia	Crackers: 0.9 Chips and flips: 5.34 Fried corn nuts: 1.7	Crackers: 0.5 Chips and flips: 0.152 Fried corn nuts: 0.1
Poland	Biscuits: 2.81 French pastry cookies: 1.65	Biscuits: 0.21 French pastry cookies: 0.275
Tunisia	Margarines: 4.47 Frying oil: 0.14	Margarines: 4.47 Frying oil: 0.24
Lebanon 2021 (current study)	Traditional dishes: 0.7 Arabic sweets: 0.5 Butter and margarines: 1.4 Biscuits, doughnuts, cake: 0.4 Cereals and breads group: 0.3 Tuna: 0.35 Chocolate and chocolate wafers: 1.26 Cooking oils: 0 Coffee and instant coffee: 0.2 Chips, nuts and seeds: 0.2 Tahina and Halawa: 0.5	Traditional dishes: 0.9 Arabic sweets: 0.6 Butter and margarines: 1.6 Biscuits, doughnuts, cake: 0.5 Cereals and breads group: 0.3 Tuna: 0.45 Chocolate and chocolate wafers: 1.3 Cooking oils: 0 Coffee and instant coffee: 0.25 Chips, nuts and seeds: 0.3 Tahina and Halawa: 0.6

## Data Availability

All data underlying the results are available as part of the article and no additional source data are required.
